# Spectral Analysis and Parameter Identification of Textile-Based Dye-Sensitized Solar Cells

**DOI:** 10.3390/ma11091623

**Published:** 2018-09-05

**Authors:** László Juhász, Irén Juhász Junger

**Affiliations:** 1 Deggendorf Institute of Technology, Faculty of Electrical Engineering, Media Technology and Computer Science, 94469 Deggendorf, Germany; 2 Bielefeld University of Applied Sciences, Faculty of Engineering and Mathematics, 33619 Bielefeld, Germany; iren.juhas_junger@fh-bielefeld.de

**Keywords:** dye-sensitized solar cell, half-textile, spectral analysis, parameter identification, equivalent circuit, black-box, grey-box, power spectral density, optimization

## Abstract

Linearized equivalent electrical-circuit representation of dye-sensitized solar cells is helpful both for the better understanding of the physical processes in the cell as well as for various optimizations of the cells. White-box and grey-box modelling approaches are well-known and they are widely used for standard cell types. However, in the case of new cell types or the lack of deep knowledge of the cell’s physic such approaches may not be applicable immediately. In this article a black-box approach for such cases is presented applied together with spectral analysis. The spectral analysis and the black-box approach were as first validated with a standard glass-based dye-sensitized solar cell and thereafter applied for the characterization of a new type of textile-based dye-sensitized solar cells. Although there are still improvement potentials, the results are encouraging and the authors believe that the black-box method with spectral analysis may be used particularly for new types of dye-sensitized solar cells.

## 1. Introduction

In praxis, it is often helpful to have a linear model for the investigated object or system. This enables among others the use of analysis techniques and definitions which are available only for linear plants. In the case of dye-sensitized solar cells (DSSC), the linearized frequency-dependent impedance is from great interest. As DSSC are generally non-linear, an appropriate linearization in the steady-state operating point is necessary. Because the linearized model is only accurate in the neighborhood of the given steady-state, it is important to choose it according to the intended use-case, which is usually in the point of the maximal energy generation.

In scope of the identification procedure an experimental measurement followed by a spectral analysis is conducted as first. Instead of the impedance spectroscopy, we propose the spectral analysis method for DSSC. Spectral analysis is not yet applied in the context with DSSC. The proposed spectral analysis method is a reasonable alternative to the standard spectroscopy methods because of its efficiency regarding the measurement duration. The standard spectroscopy methods require a separate measurement for each discrete frequency, whereas the proposed spectral estimation method processes the complete frequency band of interest based on a single measurement. The result of the spectral analysis is the non-parametric frequency transfer function (TF) for the covered frequency range. In order to get a parameterized standard TF, parameter identification is performed by using least-squares optimization procedure while using quadratic goal function.

If our goal is the optimization a process or the plant, it is important to have an accurate model in the intended steady-state working-point over the broad frequency range of interest. This is straightforward if the physical system is known in detail. However, even if the physical system is not known in detail it is still possible to perform an identification process, which is called black-box identification (BBI, [1]). If some of the elements of the system are well-known, but there are also unknown parts, grey-box identification (GBI, [1]) methods can be used. White-box linearization and parameter identification methods (WBI) are applicable if deep knowledge of the object’s physic and internal processes is available. Using the knowledge and the technological properties of the plant, in this case an equivalent model can be directly composed using known parameter values. In case of missing parameter values but known model structure, the GBI may be used. While WBI and GBI follow the bottom-up approach starting with known elements (or structure) and ending with the parameterized model of the complete system, the BBI approach is rather a top-down one. It starts with the experimental data taken from the whole system, estimates the system’s structure and it ends with the identified parameter of the structure elements.

For standard glass-based DSSC several equivalent models are published [2]. Therefore, the GBI approach is for such cells indeed a reasonable choice. In this paper, we present the BBI approach with glass-based DSSC to prove the proposed BBI concept as first. This method consists of a special form of spectral analysis and the recognition of the dominant elements in the Nyquist- and Bode-diagram followed with the proposed parameter identification procedure. The intermediate result is the parametrized frequency-transfer function, whereas the final goal of the approach is to find and parametrize a suitable equivalent electrical-circuit. As the concept of frequency transfer functions is limited to linear and time-invariant (LTI) systems, the currently proposed approach is limited to linear elements as well. The resulting parametric TF is here always a rational function and the resulting equivalent models are composed from passive linear elements (particularly R, L, and C). The extension of the proposed approach to further elements used in the modeling of DSSC (like the Warburg-element) will be the subject of our future work, whereas the concept of fractional differential equations could be helpful [3,4].

Indeed, it is not clear if the published equivalent models of glass-based DSSC are also suitable for textile-based DSSCs. Therefore, for the determination of the linear frequency-dependent impedance of the half-textile DSSC, we apply the method following the proposed BBI approach. Particularly, the BBI Spectral identification approach is applied for the characterization of dye-sensitized solar cells with counter electrodes built from electrospun polyacrylonitrile (PAN) nanofiber mat coated by different number of poly(3,4-ethylenedioxythiopene) polystyrene sulfonate (PEDOT: PSS) layers. Among others, the linearized resistance of the cells in the steady-state is determined, which shows a clear correlation with the number of PEDOT: PSS layers.

## 2. Materials and Methods

### 2.1. Measurements and Sprectral Analysis

The spectral analysis was performed for each DSSC separately in the individual steady-state point, which yields maximal active power. A sine-sweep voltage $u_{AC}\left(t\right)$ with 10% amplitude of the constant steady-state voltage and the frequency range of 1 Hz–50 kHz is applied to the DSSC. The applied voltage is recorded together with the measured current $i_{AC}\left(t\right)$ and the measurement timestamps. The measurement frequency was 100 kHz (10 µs steps). A sine-sweep generator with square frequency distribution was created and used. For the signal generation and measurements, a dSPACE MicroLabBox (MLBX, [5]) is used, which was equipped with a specially designed signal-attenuator and -converter box to achieve the best possible A/D and D/A resolution. The programming of the MLBX was performed while using MathWorks Simulink [6] in a graphical model-based way. The sine-sweep generator was implemented in Simulink. A single measurement covered 200 s and contained 5 complete sine-sweep sequences. 

### 2.2. Parameter Identification

The spectral identification was performed by the use of specially written Matlab-scripts that implemented the method of Welch for the non-parametric estimation of the TF and a quadratic goal function for the parameter identification. The periodogram size of 32768 elements with 50% overlapping is selected and Hanning window is used.

## 3. Spectral Analysis and Parameter Identification of the Linearized DSSC Model

### 3.1. Spectral Analysis Versus Impedance Spectroscopy

**Figure 1:**
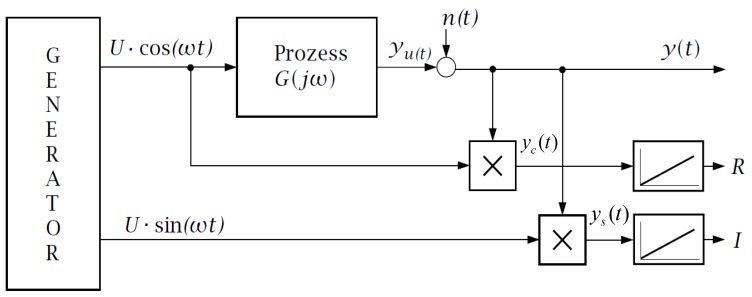
Action-diagram of correlation analysis used during impedance spectroscopy for determination of the real and imaginary parts of the transfer function (TF) for the discrete angular frequency ω.

The widely used impedance spectroscopy of DSSCs requires a separate measurement for each discrete frequency. A sine-wave voltage input is applied on top of the selected steady-state voltage to the cell and after the transient effects are faded, the amplitude and phase of the same frequency component of the established current through the DSSC is observed. In this way, the linearized complex dynamic impedance of the DSSC for the single angular frequency ω may be determined. Through repeating this procedure with different frequencies, the shape of the TF $\hat{Z}\left(j\omega \right)≅Z\left(j\omega \right)=u_{AC}\left(j\omega \right)/i_{AC}\left(j\omega \right)$ can be obtained. Commercially available equipment uses an automated method for the determination of the real and imaginary part of the single points of the TF while using the correlation analysis [1]. The action-diagram of such a system is shown in Figure 1.

In Figure 1, a signal generator outputs two signals, which are of the same, selectable frequency, and of identical amplitude but with a phase difference of *π*/2. The process output $y_{U}\left(t\right)$ is distorted by the measurement noise n(t); thus the measured output y(t) is also distorted. By choosing the measurement duration $T=k\cdot \frac{2\pi }{\omega }$ the integration yields following signals:

(1) $$R=\frac{1}{2}U^{2}\cdot k\cdot \frac{2\pi }{\omega }\cdot Re\left\{G\left(j\omega \right)\right\}+\int_{0}^{k\cdot \frac{2\pi }{\omega }}n\left(t\right)\cdot U\cdot \cos\left(\omega \cdot t\right)\cdot dt,$$

(2) $$I=-\frac{1}{2}U^{2}\cdot k\cdot \frac{2\pi }{\omega }\cdot Im\left\{G\left(j\omega \right)\right\}+\int_{0}^{k\cdot \frac{2\pi }{\omega }}n\left(t\right)\cdot U\cdot \sin\left(\omega \cdot t\right)\cdot dt.$$

In case when the disturbance n(t) is independent on the excitation, the integrals remain limited, whereas the other terms are directly dependent on k. That means, for large k, the influence of the disturbance is minimized. The real and imaginary parts of the transfer function are directly obtained for the angular frequency ω. Knowing the real and imaginary part of the TF its amplitude and phase can be also calculated. Performing further measurements with different angular frequencies, the nonparametric transfer function of the linearized system can be reconstructed.

The obvious drawback of the impedance spectroscopy is that, for obtaining the TF of a single DSSC, hundreds of measurements are necessary. Even when these measurements are performed automatically, the complete sequence takes long time and requires expensive equipment. Spectroscopy was applied in field of electrical and mechanical engineering for long time [1], however due to available and inexpensive CPU-resources nowadays, mainly the spectral analysis is used instead.

The standard spectral analysis uses the Fourier-transformation of the input and the output signal of a stable process while using a single measurement. In the case of DSSC, this means applying of AC voltage of continuously changing frequency to the DSSC additionally to the steady-state DC-voltage and the measurement of the AC-component of the established current trough the cell. Knowing the applied AC components of a DSSC cell voltage $u_{AC}$ and the measured current $i_{AC}$, the frequency-dependent dynamic cell impedance may be calculated, as follows:

(3) $$\tilde{Z}\left(j\omega \right)=\frac{ℱ\left(u_{AC}\left(t\right)\right)}{ℱ\left(i_{AC}\left(t\right)\right)}=\frac{u_{AC}\left(j\omega \right)}{i_{AC}\left(j\omega \right)}.$$

However, this method returns correct results only in case when the disturbances (e.g., measurement noise) is negligibly related to the system output. If the measurement noise n(t) is considerable, the direct use of the Fourier-transformation yields erroneous results as shown in Equation (4).

(4) $$\tilde{Z}\left(j\omega \right)=\frac{ℱ\left(u_{AC}\left(t\right)\right)}{ℱ\left(i_{AC}\left(t\right)+n\left(t\right)\right)}\neq \frac{u_{AC}\left(j\omega \right)}{i_{AC}\left(j\omega \right)}.$$

Furthermore, the leaking-effect causes a “blurred” spectrum, which further deteriorates the result. In order to determine the frequency-dependent impedance with satisfying accuracy by the direct use of the Fourier-transformation, high-end equipment, and long measurements are necessary.

### 3.2. Spectral Analysis Using the Correlation Functions

The calculation of the nonparametric frequency-response using common measurement equipment and shorter measurement periods is still possible if the periodogram-method, according to Welch [7], is applied. Using the theorem of Wiener-Khintchine [8], the non-parametric estimation of the DSSC impedance in form of a TF $\hat{Z}\left(j\omega \right)≅Z\left(j\omega \right)=u_{AC}\left(j\omega \right)/i_{AC}\left(j\omega \right)$ may be obtained using the auto-correlation of the AC current component $\phi_{i_{AC}i_{AC}}$ which is here formally the input signal and the cross-correlation of the AC voltage component (which here plays formally the role of the system output) with the AC current component $\phi_{i_{AC}u_{AC}}$.

On the one hand, the Fourier transformation of the correlation function yields the spectral power density (SPD). On the other hand, the SPD can be represented as a product of the individual signals represented in the frequency domain, see Equation (5). Thus, the non-parametric estimation of the impedance can be calculated through division of the SPD of the auto-correlation function with the SPD of the cross-correlation function.

(5) $$ℱ\left(\phi_{i_{AC}i_{AC}}\right)=S_{i_{AC}i_{AC}}\left(j\omega \right)=\;i_{AC}^{*}\left(j\omega \right)\cdot i_{AC}\left(j\omega \right)\;ℱ\left(\phi_{i_{AC}u_{AC}}\right)=S_{i_{AC}u_{AC}}\left(j\omega \right)=\;i_{AC}^{*}\left(j\omega \right)\cdot u_{AC}\left(j\omega \right)\;\hat{Z}\left(j\omega \right)=\frac{S_{i_{AC}u_{AC}}\left(j\omega \right)}{S_{i_{AC}i_{AC}}\left(j\omega \right)}.$$

The impact of the measurement noise to the calculated non-parametric impedance estimation is now significantly reduced if the measured signal is not correlated with the measurement noise. To reduce the variance of the spectral analysis, the periodogram method may be further applied [9] with an appropriate windowing of the individual periodogram data in the time domain. The information about the quality of the estimation over the used frequency range is given by the coherence-function:

(6) $$\gamma_{i_{AC}u_{AC}}^{2}\left(j\omega \right)=\frac{{\left|S_{i_{AC}u_{AC}}\left(j\omega \right)\right|}^{2}}{S_{i_{AC}i_{AC}}\left(j\omega \right)\cdot S_{i_{AC}u_{AC}}\left(j\omega \right)},\;\;0\leq \gamma^{2}\leq 1.$$

By coherence-function values near to one, the linear dependence between the input and output is dominant, thus the non-parametric estimation is good. If the coherence-function tends to zero, there is no linear dependence between the input and output and the non-parametric estimation is poor. As one may notice, this is useful information for the parameter estimation procedure.

The result of the non-parametric spectral estimation is the frequency transfer function, which is defined with complex values related to discrete frequencies. Using these values graphical representation using Nyquist or Bode diagrams can be plotted. Based on the diagrams and their characteristic elements, the appropriate TF form and finally the elements of the equivalent electrical circuits of a DSSC may be chosen.

### 3.3. Parameter Identification Procedure

The non-parametric frequency transfer-function $\hat{Z}\left(j\omega \right)$ is the basis for the estimation of the parametric TF Z(jω). During this process, we suppose the parametric TF structure and have to solve a quadratic optimization problem with some imposed restrictions.

The choice of the TF structure Z(jω) is from crucial importance, because it determines whether the optimization task succeeds or fails. The number of parameter is here also important because in case too many parameters are supposed, the system is overdetermined, and the optimization may fail because of numeric issues. In case of a too small parameter number, the parametric TF cannot describe the system behavior and the optimization task fails as well.

In case when resonance peaks are observed in the Bode-diagram, a general n-th order TF should be supposed depending on the number of the resonant peaks. For example, in case of one resonant peak, a second order transfer-function may be eligible as shown in Equation (7). Thus, *n* resonant peaks would require a 2*n*^th^ order TF, which can be decomposed into a product (or alternatively through partial fraction decomposition into a sum) of second-order TFs.

(7) $$Z\left(j\omega \right)=R\cdot \frac{b_{2}\cdot {\left(j\omega \right)}^{2}+b_{1}\cdot j\omega +1}{a_{2}\cdot {\left(j\omega \right)}^{2}+a_{1}\cdot j\omega +1}$$

Due to physical circumstances, only positive values should be allowed for the static gain R during the optimization process and furthermore only such TF parameters should be allowed which results in a stable plant. Such a second-order transfer function matches an equivalent electrical circuit consisting of RLC-elements.

If no resonant peaks are observed in the Bode-diagram, a TF consisting of simple real-valued pole-zero elements can be supposed. The number of zeros and poles may be supposed according to the number of observed “half-circles” observed in the Nyquist plot. For example, in the case when two half-circles are observed, a second-order TF with parameters $s_{p1},s_{p2},s_{z1},s_{z2}$ having the static gain R in Equation (8) can be supposed. The stability condition and the positivity of R were here also enforced from the optimization algorithm.

(8) $$Z\left(s\right)=R\cdot \frac{\left(1-\frac{s}{s_{p1}}\right)\cdot \left(1-\frac{s}{s_{p2}}\right)}{\left(1-\frac{s}{s_{z1}}\right)\cdot \left(1-\frac{s}{s_{z2}}\right)},\;Z\left(j\omega \right)=R\cdot \frac{\left(1-\frac{j\omega }{s_{p1}}\right)\cdot \left(1-\frac{j\omega }{s_{p2}}\right)}{\left(1-\frac{j\omega }{s_{z1}}\right)\cdot \left(1-\frac{j\omega }{s_{z2}}\right)}$$

Both in the case of form Equations (7) and (8) it is obvious, that the parameter R represents the linearized thermal resistance of the DSSC in the steady-state working-point, whereas in Equation (8), the relative values of the poles and zeros determine the capacitive or inductive nature of the impedance for the given angular frequency $\omega_{i}$. When vertical lines are observed in the Nyquist-diagram, they indicate the sum of static gain and pole or zero element. They can be substituted in the equivalent circuit with a series connection of R and L/C-element.

Finally, the possibility to fit the results to a specific equivalent circuit known from the literature should be checked. In cases where this was not possible, a hint for a possible equivalent circuit may be given, which fits the more general transfer function form.

The estimation of the parameter of the supposed TF-structure is performed by a quadratic optimization algorithm. One possibility is to compare the real- and imaginary-part of the non-parametric and the parametric TF for each discrete angular frequency $\omega_{i}$ and minimize the calculated quadratic error sum. The alternative way is to compare the phase and the gain differences for the building of the quadratic error sum [10]. This second option in our study showed significantly better results. The likelihood of the optimization can be improved by the multiplication of the individual error squares for discrete angular frequencies $\omega_{i}$ with the matching value of the coherence-function obtained for the same angular frequency $\omega_{i}$ in Equation (9) [10].

(9) $$e_{Zmag}=\frac{\sqrt{\sum_{n=1}^{N}{\left(\gamma_{i_{AC}u_{AC}}^{2}\left(j\omega_{n}\right)\cdot \left(\left|\hat{Z}\left(j\omega_{n}\right)\right|-\left|Z\left(j\omega_{n}\right)\right|\right)\right)}^{2}}}{\sqrt{\sum_{n=1}^{N}{\left(\left|\hat{Z}\left(j\omega_{n}\right)\right|\right)}^{2}}},\;e_{Zphase}=\frac{\sqrt{\sum_{n=1}^{N}{\left(\gamma_{i_{AC}u_{AC}}^{2}\left(j\omega_{n}\right)\cdot \left(arg\left(\hat{Z}\left(j\omega_{n}\right)\right)-\arg\left(Z\left(j\omega_{n}\right)\right)\right)\right)}^{2}}}{\sqrt{\sum_{n=1}^{N}{\left(\arg\left(\hat{Z}\left(j\omega_{n}\right)\right)\right)}^{2}}}\;e_{Z}=e_{Zmag}+w_{phase}\cdot e_{Zphase}\;$$

In Equation (9), $w_{phase}$ is a positive constant (weighting of the phase estimation), which gives a further possibility to improve the convergence of the optimization. The optimization goal is to find such parameter of the supposed TF, which results in a minimal value of the error-sum $e_{Z}$.

### 3.4. Parameter Identification Example with Glass-Based DSSC

**Figure 2:**
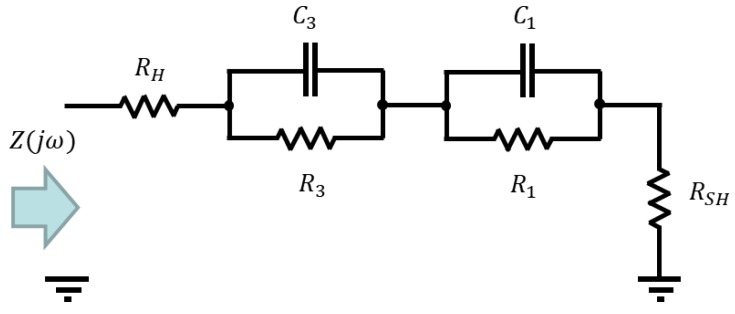
Equivalent circuit used for the parametric identification of the impedance of standard glass dye-sensitized solar cells (DSSC), according to [2].

**Figure 3:**
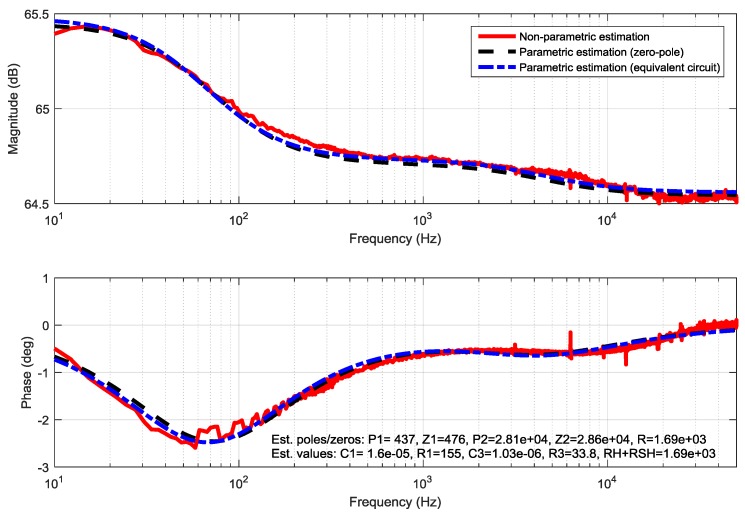
Comparison of the non-parametric and parametric TF identifications of a standard DSSC in Bode-diagram. Pole and zero values are given in (rad/s), resistance values in Ω and capacitances in F.

**Figure 4:**
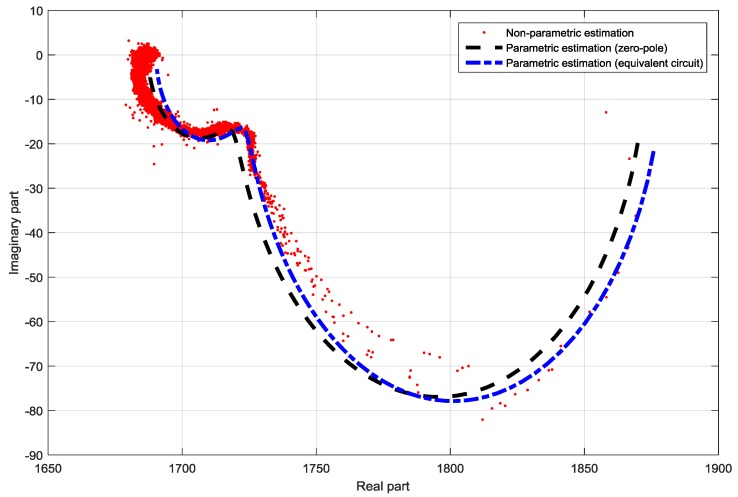
Comparison of the non-parametric and parametric TF identifications of a standard DSSC in Nyquist-diagram.

The proposed identification method was tested while using a standard DSSC with glass electrodes prepared as described in [11]. The sine-sweep signal with range of (1 Hz–50,000 Hz) was added to the constant steady-state voltage. The lower frequency boundary was set to cover all the dynamic properties of the cell, whereas the higher boundary was imposed through the used CPU-based hardware platform. From the bode-diagram of the identified non-parametric TF, it was obvious that there are no resonant peaks. The Nyquist-diagram displayed two half-circles with negative imaginary part. As this is an obvious characteristic of second-order-systems with two time constants e.g., real poles and zeros, as first the parametric identification was performed while using a second-order zero-pole element. From the Nyquist plot of the non-parametric estimation, it is furthermore obvious that it agrees with two parallel RC circuits in series with a resistor [12], which is a published equivalent circuit for standard DSSC [2]. In the following step, the structure of the supposed TF for the glass based DSSC was calculated and displayed in Equation (10) while using the equivalent circuit from Figure 2 in accordance to Thévenin’s theorem. The Bode and Nyquist diagrams of the non-parametric and parametric estimations are shown in Figure 3 and Figure 4, together with the estimated parameter.

Generally, a very good agreement between the non-parametric and parametric estimations can be noticed. This indicates the validity of the proposed method for spectral analysis and parameter estimation. Indeed, as the resistors $R_{H}$ and $R_{SH}$ are connected in series only its sum appears in the parametric TF (but not the individual ones). For this reason, it is not possible to distinguish between them during the parametric identification: only their sum can be determined.

(10) $$Z\left(j\omega \right)=\frac{\;{\left(j\omega \right)}^{2}\cdot \left(R_{H}+R_{SH}\right)\cdot R_{1}\cdot R_{3}\cdot C_{1}\cdot C_{3}+j\omega \cdot \left(\left(R_{H}+R_{SH}\right)\cdot R_{1}\cdot C_{1}+\left(R_{H}+R_{SH}\right)\cdot R_{3}\cdot C_{3}+R_{1}\cdot R_{3}\cdot C_{3}+R_{1}\cdot R_{3}\cdot C_{3}\right)+\left(R_{H}+R_{SH}\right)+R_{1}+R_{3}}{{\left(j\omega \right)}^{2}\cdot R_{1}\cdot R_{3}\cdot C_{1}\cdot C_{3}+j\omega \cdot \left(R_{1}\cdot C_{1}+R_{3}\cdot C_{3}\right)+1}$$

### 3.5. Parameter Identification Examples with Half-Textile DSSC

In the next step, the proposed spectral analysis with black-box parameter identification procedure was applied to half-textile DSSCs with nanofiber-mat based counter electrodes. The working electrodes of the DSSCs were prepared on TiO_2_-coated FTO (fluorine-doped thin oxide) glass plates (purchased from Man Solar) that were dyed with anthocyanins extracted from forest fruit tea (MAYFAIR). The counter electrodes were built on electrospun PAN nanofiber mat (prepared using the electrospinning machine “Nanospider Lab” (Elmarco, Czech Republic)) coated by one to five layers of PEDOT: PSS (CLEVIOS™ S V 4). As catalyst, a graphite layer performed by spraying was used (Graphit 33 by Kontakt Chemie). The both electrodes were put together and fixed by an adhesive tape, and the cells were filled with an iodine/triiodide based electrolyte (type 016 purchased from Man Solar). The detailed description of the cell preparation is given in [11].

**Figure 5:**
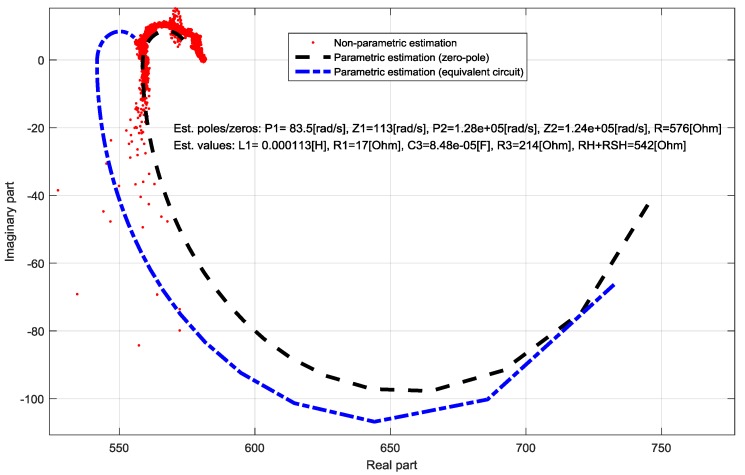
Comparison of the non-parametric and parametric TF identifications of a one-layer half-textile DSSC in Nyquist-diagram.

The measurements were taken three days after cell preparation. In this section only few representative examples will be shown and discussed. In the case of the half-textile DSSC with one layer of PEDOT: PSS a qualitative difference in the Nyquist plot related to the standard glass based DSSC is noticeable. The Nyquist plot consists of a half-circle with negative imaginary part which can be described with a parallel RC circuit [12] and a half-circle with a positive imaginary part which can be described with a parallel RL circuit [12]. Here, instead of the pure capacitive nature of the impedance known from the standard DSSC, an inductive part is also observable; this becomes dominant in high-frequency area. As there were still no significant resonance peeks in the Bode plot noticeable, for the parametric identification the pole-zero form from Equation (8) was chosen. The comparison of the Nyquist plots of the non-parametric and parametric pole-zero estimation in the frequency range from 1 Hz to 50 kHz is shown in Figure 5. The estimated parameters are displayed in the Figure 5. The agreement between the non-parametric and the pole-zero estimation is excellent.

**Figure 6:**
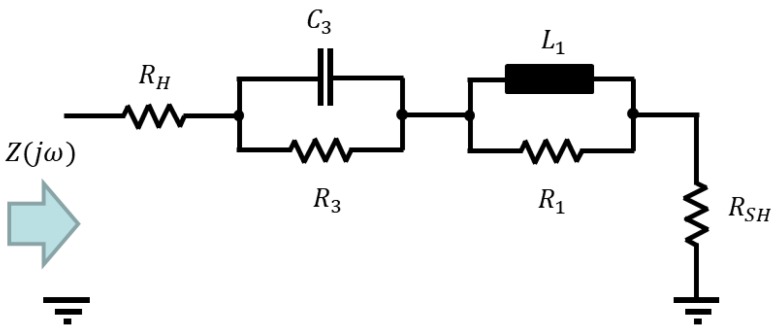
Proposed equivalent circuit for the parametric identification of the impedance of textile-based DSSC.

The effect of having inductive impedance at higher frequencies was observable by all the investigated half-textile DSSC. However, no significant resonance peaks were detected in the Bode-diagrams. Furthermore, the presence of only one R/C element was observed in all measurements for the utilized frequency range between 1 Hz and 50 kHz, e.g., only one half-circle under the ordinate in the Nyquist-plot was observable. A half-circle with positive imaginary part, which stays for inductive impedance, was always detectable. For this reason, according to the black-box method, a modified equivalent-model is supposed consisting of one parallel R/C and one parallel R/L element connected in series with the resistances, as shown in Figure 6. The equivalent impedance of the circuit is given in Equation (11).

(11) $$Z\left(j\omega \right)=\left(R_{H}+R_{SH}\right)+Z_{3}\left(j\omega \right)+Z_{1}\left(j\omega \right)=\left(R_{H}+R_{SH}\right)+\left(\frac{\frac{R_{3}}{j\omega C_{3}}}{R_{3}+\frac{1}{j\omega C_{3}}}\right)+\frac{R_{1}\cdot j\omega L_{1}}{R_{1}+j\omega L_{1}},\;Z\left(j\omega \right)=\frac{{\left(j\omega \right)}^{2}\cdot \left(\left(R_{H}+R_{SH}\right)\cdot R_{3}\cdot C_{3}\cdot L_{1}+R_{1}\cdot R_{3}\cdot L_{1}\cdot C_{3}\right)+j\omega \cdot \left(\left(R_{H}+R_{SH}\right)\cdot \left(R_{1}\cdot R_{3}\cdot C_{3}+L_{1}\right)+L_{1}\cdot \left(R_{3}+R_{1}\right)\right)+R_{1}\cdot \left(\left(R_{H}+R_{SH}\right)+R_{3}\right)}{{\left(j\omega \right)}^{2}\cdot R_{3}\cdot L_{1}\cdot C_{3}+j\omega \cdot \left(R_{1}\cdot R_{3}\cdot C_{3}+L_{1}\right)+R_{1}}$$

As it can be noticed from Figure 5, the parametric estimation of the proposed equivalent circuit elements shows a slight difference that is related to the parametric estimation while using zero-pole elements. Although this difference indicates that there are still secondary effects that are not modelled with the proposed equivalent circuit, the main properties of the textile-based DSSC, especially the inductivity are described with the proposed equivalent model.

**Figure 7:**
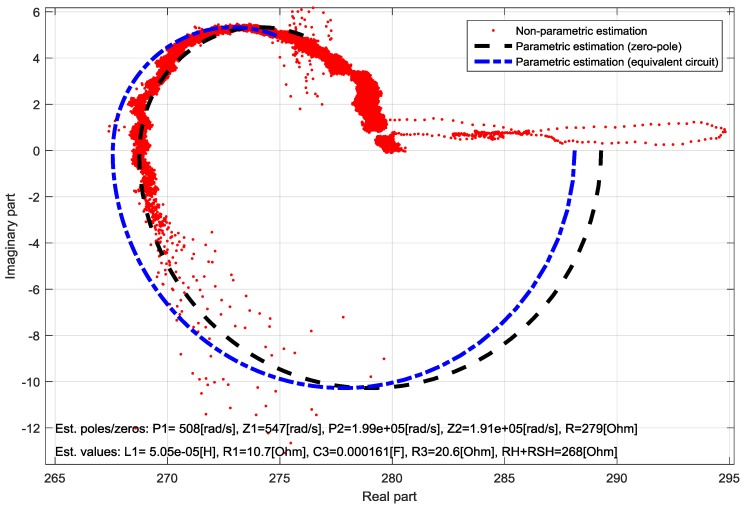
Comparison of the non-parametric and parametric TF identifications of a three-layer half-textile DSSC in Nyquist-diagram.

Similar results are obtained for the textile DSSC with three PEDOT: PSS layers, where the differences between the non-parametric and parametric estimations are also small, despite of the obviously high measurement noise. The Nyquist-diagram of the identified impedance with the obtained parameter for this case is shown in Figure 7.

## 4. Identification Results Summary

For all the investigated cells, both pole-zero as well as equivalent-circuit based parameter identification are performed. The agreement between the two identification methods is good with slight differences. In Table 1 the summary of the parameter identification sequence is given, e.g., the obtained parameter for the different half-textile DSSCs. From the point of view of the potential user, the most important element may be the cell equivalent dynamic resistance at the steady-state working point, because it limits the generated energy flow-rate. This is the parameter R from Equation (8) or equivalently the sum $R_{eq}=R_{H}+R_{SH}+R_{3}$ from Equation (11).

According to the results that are shown in Table 1, there is a strong correlation noticeable between the number of layers and the linearized resistance of the half-textile DSSC in the steady-state working point. With the enlarging the number of layers up to four layers, the dynamic resistance (R or equivalently $R_{eq}$) decreases. The DSSC with five layers of PEDOT: PSS has, however an increased resistance related to the four-layer one. This is in agreement with the results in [11], where the efficiency of the cells with five layers of S V 4 is slightly decreased when compared to the efficiency of cells with four layers of S V 4, and furthermore the I-U curve of the cells with five layers of S V 4 lies lower than the I-U characteristics of cells with four layers. The reason for the increase of the resistance of cells with five S V 4 layers is, however, unclear. To exclude that it is a result of a systematic error, the experiment will be repeated in the future. An inverse tendency can be observed in the case of the identified cell inductivity $L_{1}$.

## 5. Discussion

Spectral analysis and parameter identification of half-textile dye-sensitized solar cells according the Black-Box approach are presented. The identification approach was first tested with a standard glass-based DSSC and thereafter applied to DSSCs with textile-based counter electrodes that were coated by a different number of PEDOT: PSS layers. A strong correlation between the number of layers and the linearized resistance of the half-textile DSSC was observed.

Although there are still improvement potentials left, the authors believe that the presented spectral analysis together with the proposed black-box method can be successfully applied by new types of DSSC where the deep knowledge of the processes is not established yet. In our future work, we will concentrate on further development and extensions of the proposed equivalent circuits in order to describe the higher-order effects. For this reason, on one hand, the extending of the frequency range for the spectral analysis up to 1 MHz by means of direct programming of the embedded FPGA of the MLBX is scheduled. On the other hand, the authors will continue the work toward supporting further elements which are used by modeling of DSSCs. The concept of TF based on fractional differential equations may be helpful for this task.

## Figures and Tables

**Table 1:** Obtained parameters for half-textile DSSC cells with one to five layers of PEDOT: PSS in the counter electrode by spectral identification.

Cell Type	*R*^1^ (Ω)	*R*_eq_^2^ (Ω)	*L*_1_^2^ (mH)	*R*_1_^2^ (Ω)	*R*_3_^2^ (Ω)	*C*_3_^2^ (µF)
Single layer	575.91	755.53	0.112	16.95	213.65	84.83
Two layers	528.58	529.60	0.410	0.081	22.83	447.99
Three layers	279.14	288.14	0.050	10.72	20.59	161.31
Four layers	130.47	165.70	0.007	3.963	36.76	71.451
Five layers	242.43	253.66	0.016	6.744	17.13	144.27

^1^ From the pole-zero TF estimation. ^2^ From the equivalent-circuit TF estimation.
